# British Institutions for the Care of the Inebriate.—VI

**Published:** 1904-01-02

**Authors:** 


### BRITISH INSTITUTIONS FOR THE CARE OF THE INEBRIATE.—VI.

By Our Special Medical Commissioner.

(*Continued from Page* 160.)

### BUNTINGFORD HOUSE RETREAT.

The thought of yesterday lives in the activity of to-day. Mediæval conceptions still linger and profoundly influence our conduct in combating disease. Past methods yet mould much of present-day practice. This is particularly the case in regard to the control of the inebriate. We have been slow to realise that such hygienic conditions as were necessary for the maintenance of well-being in the normal subject were also absolutely essential for the restoration of the afflicted. Slowly but surely rational views have gained ground and are now exerting very considerable directing force. The application of hygienic principles to the management of the mentally deranged, and the enforcement of conditions, making for healthy life on the physically disordered, have proved that natural methods are rich in restorative influences.

Until recently the inebriate has often been treated mainly as a moral delinquent, fit for punishment, and needing repression; and much in customary methods, nominally aiming at restoration, have been allowed to exert an oftentimes seriously prejudicial action.

The time, however, is arriving when the "retreat," with its barred doors and closed windows, and environment confining to body and fettering for mind, must make way for the "sanatorium," where the inebriate shall be brought under the action of natural influences which shall upbuild bodily vigour, strengthen mental action, and fortify moral powers.

The history of the evolution of institutions for the care of the insane, is the record of a passage from barbarism to rational and disciplined life. But it must be admitted that even to-day many of the so-called "retreats" for inebriates have still many elements of barbarism pertaining to them. In only too many instances the convenience of friends seem to have been studied rather than the comfort and suitable treatment of the patients.

For the successful treatment of the inebriate it is manifest that conditions making for the establishment and maintenance of the hygienic life are essential.

The environment must be such as will allow of the full development of physical powers, the rehabilitation of the will, and the re-creation of the oftentimes blurred moral qualities.

The inebriate in most instances is the subject of distinct tissue impairment; and instability or paralysis of the will and deterioriation of the latest acquired and most necessary elements in the human upbuilding are always conspicuous.

In the re-creation of inebriates it is most desirable that they should be given opportunities for living an outdoor life which may restore the physical frame, and be trained sympathetically but systematically, and with well directed discipline, in some forms of useful and yet attractive work which shall aid in recruiting mental power and enforcing moral control.

These principles are apparently the guides directing the conduct of Butingford House Retreat.

The Retreat is pleasantly situated near the quaint village of Buntingford at an elevation of 400 feet in a healthy, secluded, and picturesque district of Hertfordshire.

It is within a quarter of a mile of Buntingford Station (G.E.R.), which can be reached in about 1½ hours from Liverpool Street.

Buntingford House was formerly the local grammar school. The ancient foundation date from the time of James I., and was situated in the village, but new buildings were erected in 1878, and these by some simple modifications have been converted to their present purpose. The medical superintendent and licensee, Dr. G. Melville Smith, occupies the portion of the establishment which formerly was the headmaster's house.

The lower part of the old main school-room forms a good dining hall. There is also a recreation and concert room, a reading room well supplied with books, and an excellent billiard table.

Sleeping accommodation is provided in dormitories and cubicles, but there are also a few private bedrooms.

The establishment is heated by hot water, and there is a very satisfactory electric light installation. The lavatories and baths might be modernised with advantage.

The grounds, 10½ acres in extent, contain cricket ground, tennis courts, recreation grounds, a poultry farm, flower, vegetable and fruit gardens, lawns, cycle track, and numerous paths.

There are also good workshops where joinery, woodcarving, bookbinding, and the like may be carried on. A well-equipped photographic dark room is provided.

With the opportunities available, much good work should be accomplished in securing the willing co-operation of the inmates in the work of the garden, the poultry farm, and the workshops. For the success of such measures, however, tactful and experienced teachers are necessary who shall have endless patience and an enthusiasm for their oftentimes peculiarly discouraging work.

The Retreat is intended for gentlemen willing to undergo detention and treatment either under the Acts or as private patients.

Treatment appears to be directed in accordance with sound principles. There is a well stocked dispensary. An electric light bath is found useful for morphia cases. But chief reliance is placed in the strict maintenance of total abstinence, with open-air life, and regulated occupation.

Although the medical superintendent has power to enforce work for patients "under the Act," we understand there is but little need to use anything like compulsion, as the patients are usually willing and even anxious to take up some kind of occupation. Some six hours a day are supposed to be spent in work, during which smoking is not allowed. Much time is allowed for recreation, and outdoor pursuits are particularly encouraged. A short golf course is confined to the grounds, but four drives of considerably over 200 yards each are obtainable.

We have carefully studied the regulations and orders for the conduct and management of the establishment and find them satisfactory.

The institution is capable of accomplishing much good work. At present it needs modernising in several ways. There is ample opportunity for extension. The outdoor work should be developed, and the patients might well be trained in the various branches of gardening and cultivation of fruit and flowers. The poultry farm will no doubt in time be considerably enlarged. The institution is in course of development, but with wise direction and careful management success should be assured.

The Retreat is duly licensed under the Inebriates Acts, 1879–99. It is, however, to all intents and purposes a private establishment.

We think it would be well if all such institutions for inebriates could in addition to the resident medical superintendent, who would of course usually be the licensee, be regularly visited by some well-known physician of consultant rank. The in-coming at regular periods of an outside medical adviser would, we believe, be advantageous to the patients and generally beneficial to the institution.

On the occasion of our visit to Buntingford Retreat, we were unable to examine any of the patients, but we were given to understand that the results in many cases are favourable. The Retreat is licensed for the reception of 35 male cases.

Patients are admitted on the following terms:—For a quarter of 13 weeks, with private bedroom, 52s. 6d. a week; with cubicles, 42s. a week; and with dormitory accommodation for cases who cannot afford more, 31s. 6d. per week. Private patients and drug cases are charged half-a-guinea a week extra. Dormitory cases are only taken for a minimum period of six months. Two cases can be accommodated in the medical superintendent's house at five guineas a week.

These terms include board and residence and medical attendance. Laundry charges are £1 1s. per quarter payable in advance.

Should any patient leave the Retreat from any cause at any period during a quarter the payment in advance for the remainder of that quarter will be forfeited.

